# Supplementary figures and images for: Lipid Profile Rather Than the LCAT Mutation Explains Renal Disease in Familial LCAT Deficiency
Source: J Clin Med. 2019 Nov 3;8(11):1860. doi: 10.3390/jcm8111860 (PMC6912718; doi:10.3390/jcm8111860)

Lipids

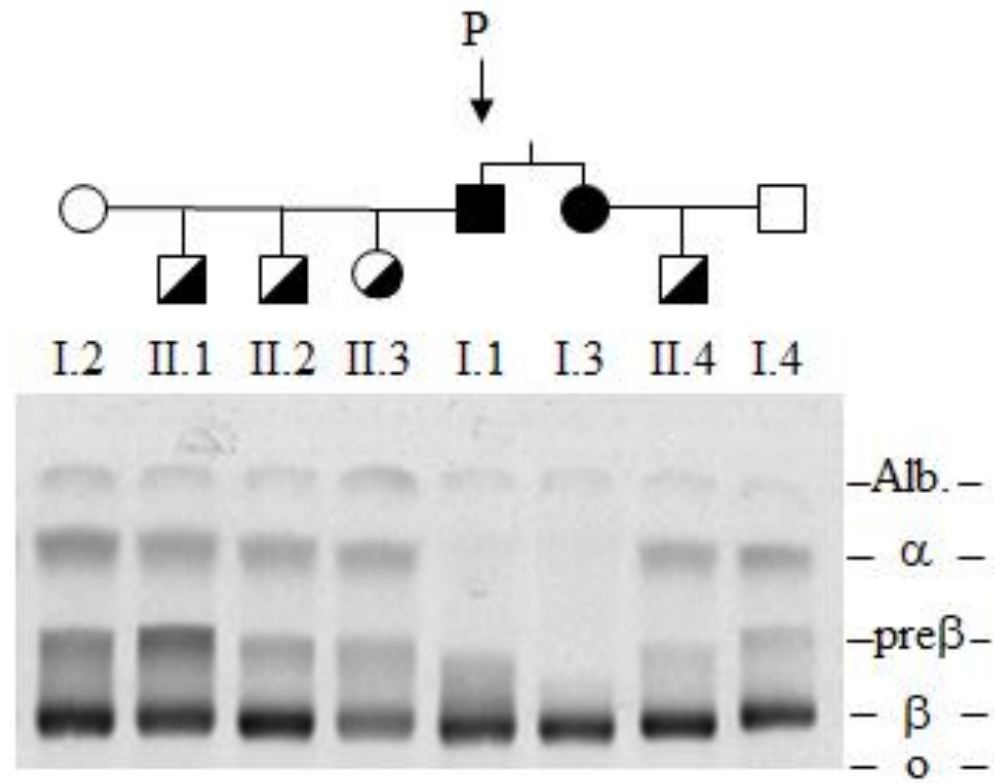

Supplement: Supplementary file 1 [file jcm-08-01860-s001.zip › Supplemental Figure 1.pdf]

Control 1 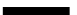 CTR  
Control 2 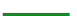 CTR  
Control 3 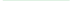 CTR  
Second case 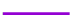 LCAT  
Third case 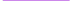 LCAT

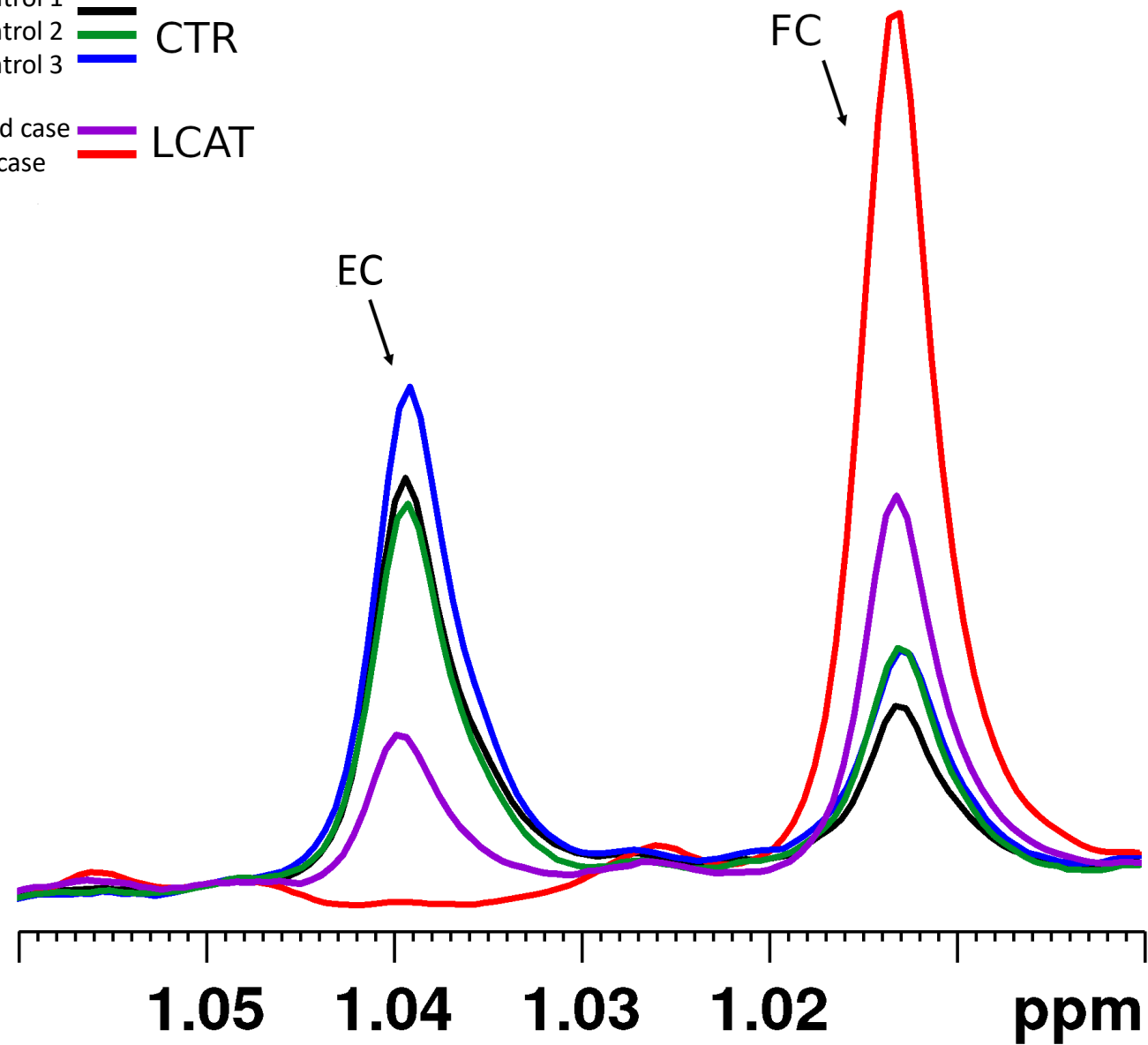

Supplement: Supplementary file 1 [file jcm-08-01860-s001.zip › Supplemental Figure 2.pdf]
